# Strategy adoption depends on characteristics of the instruction, learner, and strategy

**DOI:** 10.1186/s41235-019-0158-3

**Published:** 2019-03-21

**Authors:** Sarah A. Brown, David Menendez, Martha W. Alibali

**Affiliations:** Department of Psychology, University of Wisconsin – Madison, 1202 W Johnson Street, Madison, WI 53706 USA

**Keywords:** Strategy change, Mathematics learning, Problem solving, Feedback, Confidence, Need for cognition

## Abstract

Why do people change their strategies for solving problems? In this research, we tested whether negative feedback and the context in which learners encounter a strategy influence their likelihood of adopting that strategy. In particular, we examined whether strategy adoption varied when learners were exposed to a target strategy in isolation, in conjunction with their own current strategy, and in conjunction with another novel strategy. We also investigated the roles of individual differences, including learners’ need for cognition and their confidence in their current strategies. In Study 1, undergraduate participants who encountered a target strategy in isolation were more likely to adopt it than participants who encountered it in the context of their own current strategy. Negative feedback, low confidence, and high need for cognition also predicted greater adoption. In Study 2, we examined whether rates of strategy adoption depended on the target strategy itself. Indeed, participants were more likely to adopt one strategy than the other, and the effects of feedback also varied across strategies. Individual differences—need for cognition and confidence—also influenced patterns of strategy adoption. These results suggest that strategy adoption depends on the confluence of many factors, including the context in which a target strategy is introduced, characteristics of the learner, and characteristics of the strategy itself.

## Significance

When people learn about a new strategy, they sometimes try out the new strategy, but they sometimes do not. Understanding why exposure to new strategies can have different effects on strategy adoption is highly relevant for understanding cognition and for improving educational practice. In this work, we examine several factors that might predict whether learning about a new strategy leads to strategy change. Learners were more likely to adopt the new strategy if they received negative feedback, and they were less likely to adopt it if they encountered it in the context of their own current strategy. Individual differences also mattered: learners high in need for cognition, which is the tendency to engage in effortful processing, were more likely to adopt the new strategy than learners low in need for cognition. Similarly, learners who were less confident in their current strategies were more likely to adopt the new strategy than were learners who were highly confident in their current strategies. The specific strategy also mattered; one novel strategy was adopted by many learners, and the other by relatively few. Thus, this research suggests that many factors affect strategy change; simply teaching a learner about a new strategy does not ensure that he or she will adopt it. Instead, characteristics of the instruction, the learner, and the strategy itself are important predictors of strategy adoption.

## Background

People often solve mathematics problems incorrectly or inefficiently when they first encounter them. Over time, people replace those incorrect and inefficient strategies with newer and better ones, and this shift often leads to improvements in performance (e.g., Dean & Malik, 1986; Lemaire & Callies, 2009; Siegler & Jenkins, 1989; Torbeyns, De Smedt, Ghesquière, & Verschaffel, 2009). This process is not unique to mathematics; there is evidence from a wide range of problem domains that strategy use improves with experience and expertise (e.g., locomotion, Adolph, Vereijken, & Denny, 1998; proportional reasoning, Fujimura, 2001; memory, Schneider, Kron-Sperl, & Hünnerkopf, 2009; scientific thinking, Boncoddo, Dixon, & Kelley, 2010; Feil & Mestre, 2010; Siegler & Chen, 2008). Given the importance of strategy change in learning and instruction, it is crucial to understand factors that encourage learners to adopt new problem-solving strategies.

Previous research has identified a wide array of factors that can influence learners’ likelihood of adopting a new strategy. Some of these factors concern the features of the instruction learners receive, such as feedback about existing strategies (Fyfe & Rittle-Johnson, 2017) and exposure to alternative strategies (Brown & Alibali, 2018). Other factors concern characteristics of the individuals themselves, including both stable individual differences and more transitory characteristics, for example, whether learners have relevant prior knowledge (Fyfe & Rittle-Johnson, 2016b), and whether they are generally interested in trying new things. In previous work, we have argued that strategy change can be conceptualized in terms of the interactions of multiple factors at different levels of analysis (Alibali, Brown, & Menendez, 2019). A dynamic perspective on strategy change can provide a richer, more comprehensive view of this process, which may lead to greater insights into what makes learners decide to try new strategies for solving problems. Following this perspective, in this research, we examine the roles of specific features of the instructional context as well as individual differences and strategy differences in strategy adoption.

## Features of instruction

Many features of instruction may encourage learners to change their strategies. These include whether the learner receives instruction that provides new strategies (e.g., Brown & Alibali, 2018; Matthews & Rittle-Johnson, 2009), whether the learner receives feedback about existing strategies (e.g., Fyfe & Brown, 2018a; Fyfe & Rittle-Johnson, 2017), whether critical information is highlighted (e.g., Alibali, Crooks, & McNeil, 2018; Joh & Spivey, 2012), and whether learners work with others or alone (e.g., Fawcett & Garton, 2005; Gutiérrez, Brown, & Alibali, 2018). In this research, we consider the effects of two experiences that are common in educational settings: explicit negative feedback and exposure to alternative strategies.

### Explicit negative feedback

Intuitively, it seems likely that telling learners that their answers or strategies are incorrect via direct, negative feedback should reduce their confidence in their strategies and encourage them to attempt new strategies. In line with this intuitive expectation, meta-analyses show that, in many cases, feedback has positive effects on learning (Hattie & Timperley, 2007; Kluger & DeNisi, 1996). However, feedback can also have negative effects; in Kluger and DeNisi’s (1996) meta-analysis, approximately one-third of the effect sizes were negative. One possible explanation, suggested by Kluger and DeNisi (1996), is that feedback may be negative when it leads to a focus on the learner, rather than the problem (Fyfe & Rittle-Johnson, 2016b). In the present study, feedback was provided privately, by asking learners to compare their solution to the correct solution, so it did not focus attention on the learners themselves. For this reason, we predicted a positive rather than a negative effect of feedback. Specifically, we predicted that receiving explicit negative feedback would lead to more strategy change compared to receiving no explicit feedback.

Prior work has also shown that the effects of feedback are often moderated by learner characteristics, such as task expectations or level of prior knowledge (e.g., Fyfe & Brown, 2018b; Fyfe & Rittle-Johnson, 2016a, 2016b). In this research, we also expected that the role of feedback might depend on learner characteristics, as we discuss below.

### Exposure to alternative strategies

When learners are exposed to new strategies, either via direct instruction or by observing others solving problems, they might choose to try out those strategies. Indeed, previous work has shown that children who are exposed to different strategies for solving a problem are more likely to shift their strategy use than are children who are not exposed to new strategies (Brown & Alibali, 2018).

It remains unclear, however, in what ways the *context* in which a strategy is presented affects the likelihood that a learner will adopt that strategy. Consider two possible scenarios. In both, a learner sees two other learners solve a problem and thus observes two possible strategies for solving the problem. In one case, both strategies are new to the learner. In the other case, one strategy is new to the learner and the other strategy is the learner’s own current strategy. Exposure to these two sets of strategies may have different effects. When learners discover that other people share their current strategy (as in the second case), they may assume that they should *maintain* that strategy. In other words, exposure to one’s own strategy may serve as indirect *positive* feedback, and it may therefore hinder strategy change.

In this study, we investigate how exposure to current and new strategies influences whether learners adopt new strategies. We hypothesized that learning that another person uses the same strategy may be a form of implicit positive feedback about the validity of one’s current strategy and may serve to reinforce that strategy. Therefore, we predicted that learners would be less likely to adopt a target strategy when it is presented in the context of the learners' current strategies.

In addition, we hypothesized that the effect of exposure context might be moderated by negative feedback. We expected that learners would be more likely to adopt a new strategy if they received explicit negative feedback about their current strategy, but that this effect would be stronger when learners were not also exposed to their current strategy. When learners receive both explicit negative feedback and implicit positive feedback, the implicit positive feedback might neutralize or dampen the effect of the explicit negative feedback.

## Learner characteristics

Characteristics of learners themselves might also influence their likelihood of strategy change, either directly or by affecting how they respond to external, contextual factors. Both stable individual differences and more temporary states may be relevant. In this paper, we consider one stable characteristic, namely learners’ *need for cognition*, and one more transitory characteristic, namely, learners’ confidence in their current strategies.

### Need for cognition

Need for cognition is the inclination to engage in effortful cognitive activity and to derive enjoyment from doing so (Cacioppo & Petty, 1982). It has high test-retest reliability, suggesting that it is a stable individual difference (Sadowksi & Gülgöz, 1992). Several studies have shown that need for cognition is associated with academic performance (Sadowksi & Gülgöz, 1992, 1996). Specifically, people who score high on measures of need for cognition tend to perform better on mathematical tasks (Kokis, Macpherson, Toplak, West, & Stanovich, 2002; West, Toplak, & Stanovich, 2008). We hypothesized that people with greater need for cognition would be more likely to adopt new strategies for a mathematical task than people with lower need for cognition, because high need for cognition is associated with mathematics performance. In addition, people high in need for cognition may be generally more interested in trying out new methods for solving problems. We also hypothesized that need for cognition might moderate the effects of feedback and exposure to alternative strategies. However, given the lack of prior work on this issue, we do not advance a specific, directional hypothesis.

### Confidence in current strategies

Learners’ confidence in their current strategies could also influence whether they tend to attempt new strategies for solving a problem. Learners who are highly confident in their current strategies might be unlikely to try a new strategy because they believe that they are already solving the problem correctly. In contrast, learners who lack confidence in their current strategy might be more inclined to explore alternatives. It is also possible that learners’ confidence in their answers might moderate the effect of feedback on learning. This prediction follows from research on the *hypercorrection effect*, which is the tendency for people who receive negative feedback to be more likely to correct their mistakes if they were highly confident in their initial answers than if they were less confident in their initial answers (Butterfield & Metcalfe, 2001, 2006; Fazio & Marsh, 2009).

In the present study, we asked participants to rate their level of confidence in their strategies on each problem. We hypothesized that participants’ level of confidence in their pretest strategies would be negatively related to strategy change overall, and that it might moderate the effects of negative feedback.

## Characteristics of strategies

Characteristics of the strategies themselves might also influence learners’ likelihood of adopting those strategies. Learners may be less willing to try strategies that involve many steps or that are difficult to understand. Little work has investigated how participants evaluate strategies and whether these evaluations affect participants’ willingness to adopt strategies. In one study, undergraduates evaluated the type of problem used in the present studies. The researchers found that the undergraduates based their evaluations on two factors: efficiency and intuitiveness (Brown, Menendez, & Alibali, 2018). We suggest that these differences among strategies might affect participants’ likelihood of adopting a particular strategy. For example, people might be unwilling to adopt a new strategy if they view it as complex or unintuitive.

## Current studies

This research investigates how learners’ patterns of strategy change vary depending on two aspects of the instruction they receive—the presence or absence of explicit negative feedback and the context in which the target strategy is presented—and two characteristics of the learners themselves—need for cognition and confidence in their pretest strategies. We consider these factors for two different strategies so that we can evaluate whether patterns of change are similar or whether characteristics of the specific strategies matter.

We address these issues in the domain of constant change problems. These problems present a description of a situation in which a rate changes constantly during a time interval of a specified length. For example, one problem read, “Water is piped into a tank for a period of 7 minutes. The rate at which it is piped increases steadily over the interval from 10 liters per minute to 52 liters per minute. How many liters are piped into the tank over the 7-minute interval?”

These problems are well-suited for studying strategy change because there are multiple strategies for finding the correct answer. The most common strategy that undergraduate students use to solve such problems involves finding the constant by which the rate changes (in the problem presented above, 7), finding the relevant quantity for each unit of time (in the problem above, 10 liters in the first minute, 17 liters in the second minute, and so on), and then adding these numbers together to find the total. We will refer to this strategy as the *summation* strategy because it involves summing together a set of discrete values. Another possible strategy is the *area* strategy, which involves drawing a model of the problem and finding its area using either geometric formulas or integration. Finally, the *Gauss* strategy^1^ involves adding the initial and final rates and multiplying that sum by half of the number of units of time (e.g., for the problem presented above, $\left(10+52\right)\times \frac{7}{2}$).

Given that the summation strategy is the most common strategy among college students who have not received instruction about constant change problems (Riggs, Kalish, & Alibali, 2015), we expected that the majority of our participants would use this strategy at pretest. This allowed us to create exposure conditions that would—for the majority of participants—involve exposure to purely novel strategies as well as to their current strategy.

In Study 1, we compared strategy change when participants were exposed to no novel strategies (control), to a single novel strategy in isolation (i.e., the area strategy on its own), to the novel strategy paired with the participants’ current strategy (i.e., area and summation), or to two novel strategies (i.e., area and Gauss). This design allowed us to compare the likelihood of strategy change when (a) participants learned about one novel strategy paired with the information that another learner shared their current strategy to (b) a condition in which the participants learned only about the novel strategy. This comparison controls for the number of *novel* strategies to which participants are exposed because in each of the relevant conditions, participants were exposed to only one novel strategy: the target area strategy. In addition, this design allows us to compare the likelihood of strategy change when (a) participants learned about a novel strategy paired with the information that another learner shared their current strategy to (c) a condition in which the participants learned about two novel strategies. This comparison controls for the total number of strategies—novel or familiar—to which the participants were exposed (two). In Study 2, we tested participants in additional conditions with a different target strategy, the Gauss strategy. Specifically, we examined the likelihood of strategy change when participants were exposed to a different novel strategy in isolation (i.e., the Gauss strategy on its own) or when paired with participants’ current strategy (i.e., Gauss and summation).

## Study 1

### Method

#### Participants

We recruited 320 undergraduate students from a large Midwestern University to participate in exchange for extra credit in their introductory psychology course. Of the 320 participants, 182 identified as female and 123 identified as male; 15 chose not to report their gender. Additionally, 204 described themselves as white, 57 as Asian or Asian-American, 12 as Black or African-American, 14 as Hispanic or Latinx, and 9 as bi- or multi-racial; 24 chose not to report race or ethnicity. The average age of the participants was 19.11 years (*SD* = 1.02).

Thirteen (4%) participants were excluded from analysis due to experimenter error and 7 (2%) were excluded because they did not follow directions (i.e., they looked back to previous pages in the packet despite instructions not to do so). Because our research question concerned how people react when they learn that another (fictitious) student used the same strategy that they did, we planned to include only participants who used the summation strategy at pretest. On this basis, we excluded 79 participants from analysis (25%) because they used a strategy other than the summation strategy on the pretest (information about how strategies were coded is provided below). This ensured that our manipulation, which was intended to expose participants to either a novel or a familiar strategy, did indeed utilize strategies that were novel or familiar to the participants. Thus, the study sample consisted of 221 participants.

#### Design

The study used a 2 (feedback: no feedback or negative feedback) × 4 (strategy exposure context: control, target-in-isolation, target-with-own-strategy, target-with-other-novel-strategy) between-subjects design. Participants were randomly assigned to one of the eight conditions.

#### Materials

Packets distributed to the participants contained a pretest, a feedback intervention, an interest in exposure measure, an exposure intervention, a posttest, and a series of questionnaires. The order of these sections was the same for all participants. The pretest, interest in exposure measure, posttest, and questionnaires were the same for all participants, regardless of condition. The feedback intervention and exposure intervention sections varied by condition.

#### Procedure

Participants completed the study in one experimental session with up to two other participants. The experimenter told participants that they would complete a packet containing math problems and questionnaires and instructed the participants to complete the pages in order, without looking ahead or looking back. All experimental manipulations were implemented within the packets. Participants were randomly assigned to conditions, and the experimenter handed out the packets accordingly. Participants were given up to 45 min to complete the packet. In addition to the packet, each participant received an answer sheet on which they were instructed to record their answers. The answer sheet allowed the participants to see their answers to previous problems. This was necessary as the participants in the feedback condition had to compare the “correct” answer provided in the feedback section of the packet to their own answer to determine whether they had reached the correct answer. The procedure was identical for all participants regardless of condition.

##### Pretest

The pretest consisted of the following two constant change problems:

- There is a bookshelf with 6 shelves. The number of books on each successive shelf from top to bottom increases by a constant from the number of books on the shelf above it. There are 10 books on the first shelf and 40 books on the sixth shelf. How many books are there in total on the 6 shelves?
- A tree grows for a period of 7 weeks. The rate at which it grows increases steadily over the interval from 4 mm per week to 46 mm per week. How many millimeters does the tree grow over the 7-week interval?

The problems appeared in a fixed order that was consistent across conditions. Each problem appeared on a separate page, so that only one problem was visible at a time. Each problem was followed by a question that prompted the participants to rate how confident they were that they had solved the problem correctly on a scale from 1 to 5, with 1 representing “I’m sure I did it wrong” and 5 representing “I’m sure I did it right”.

##### Interventions

The two interventions occurred between the pretest and the posttest problems. The feedback intervention always came before the exposure intervention because we were interested in whether the feedback manipulation would influence how participants responded to the exposure intervention.

**Feedback intervention**

This section varied based on the condition to which participants were randomly assigned. Participants who were randomly assigned to receive explicit negative feedback saw a page that read, “The answer to the previous question was 188. Did you get the correct answer to the previous problem?” Participants circled either “yes” or “no”. We wanted to guarantee that all participants in the feedback condition received *negative* feedback. For this reason, we chose to use the number 188, as it was approximately halfway between two common answers people get when using the summation strategy.^2^ The actual correct answer to the problem was 175. Thus, all participants in the feedback condition were told that an incorrect answer was correct, and they were asked to compare this (incorrect) answer to their own answer. For some participants, their conclusion (that their own answer was incorrect) was accurate (because their answers were in fact incorrect, even though they were not 188). For other participants, their conclusion (that their answers were incorrect) was inaccurate because they had actually provided the correct answer of 175. To reduce any potential negative effects of this mild deception, we thoroughly debriefed participants at the end of the study.

Participants who were randomly assigned to *not* receive feedback were given no information about the correctness of their answer or the validity of their strategy. They simply proceeded directly from the pretest to the interest in exposure measure.

**Interest in exposure measure**

Next, participants were asked to report how interested they would be in reading about how other students solved constant change problems. The question read, “How interested are you in reading about how other students solved this kind of problem?” Participants responded using a 5-point Likert scale that ranged from “not interested at all” to “extremely interested”. This measure always came immediately before the exposure intervention. In the feedback conditions, this measure occurred immediately after the feedback. In the no-feedback conditions, this measure occurred after the pretest. Participants’ reports of interest in exposure to other strategies were not related to strategy adoption, so we do not consider this measure further.

**Exposure intervention**

The exposure intervention came next. This intervention consisted of two new constant change problems with worked examples of solution strategies (or not, in the control condition). These two example problems were consistent across conditions; however, the accompanying example strategies varied depending on condition.

In the *control* condition, the two example problems were presented without example strategies; instead, participants answered two questions about the content of the problems (e.g., “For how many minutes is fuel pumped into the tank?”).

**Fig. 1:**
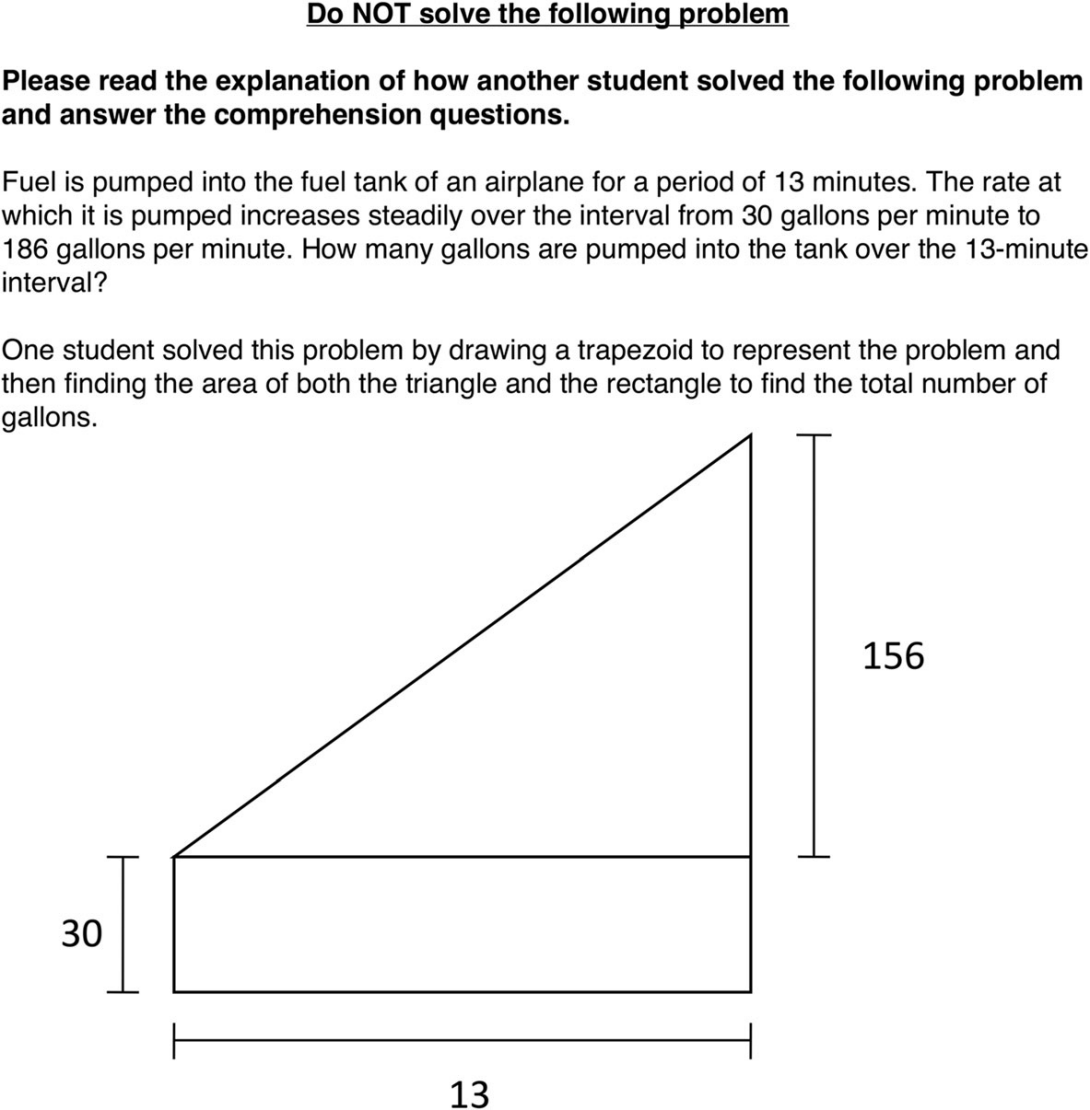
Description of the target area strategy from the exposure section of the packet

In the other three conditions, one of the two example problems was accompanied by a brief worked example of the target *area* strategy. This example was a brief description of how “another student” solved the problem. This description was accompanied by a diagram (see Fig. 1). The strategy was not named, nor was the student using the strategy described in any way. After the example, the participants answered two comprehension questions regarding the example strategy (“what number did the student use as the height of the triangle?” and “what number did the student use as the base of the trapezoid?”).

**Fig. 2:**
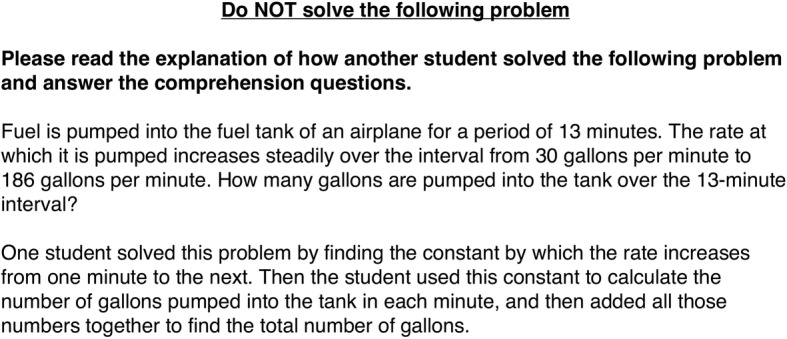
Description of the summation strategy from the exposure section of the packet

**Fig. 3:**
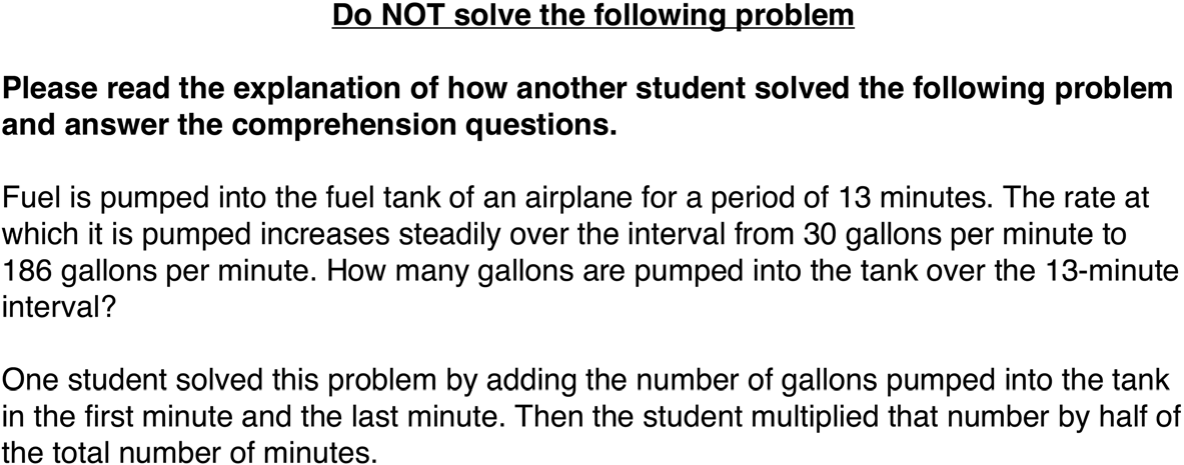
Description of the Gauss strategy from the exposure section of the packet

The three non-control exposure conditions differed in the information that accompanied the other example problem. In the *target-in-isolation* condition, the other example problem was accompanied by the same control questions used in the control condition, without a worked example strategy. In the *target-with-own-strategy* condition, the other example problem was accompanied by a worked example of the summation strategy (i.e., the strategy that participants in the study sample used at pretest; Fig. 2). In the *target-with-other-novel-strategy* condition, the other example problem was accompanied by a worked example of the Gauss strategy, another method people sometimes use to solve constant change problems (Fig. 3).

The order of the problems was constant for all participants regardless of condition, but the order in which participants read about the different strategies was counterbalanced across participants in all conditions. For example, half of the participants who were randomly assigned to the target-with-own condition were first exposed to the target area strategy and then the summation strategy, and the other half were exposed to the strategies in the reverse order.

##### Posttest

The posttest consisted of three constant change problems. As in the pretest, after each problem, participants were asked to rate their confidence in the strategy they used. After the posttest, all participants who read about one or more strategies (i.e., all participants except those in the control condition) were asked to report whether any of the strategies that they read about were the same as the strategy they had used at pretest. Participants could circle “yes”, “no”, or “I’m not sure”. This question was confusing for many participants, and we do not analyze it further.

##### Questionnaires

Finally, all packets included a series of questionnaires. These included (1) a set of items about participants’ interest in and attitudes about math, (2) the need for cognition short form (Cacioppo, Petty, & Kao, 1984, p. 307), and (3) a questionnaire about participants’ math history and demographics. As part of the demographic questionnaire, participants reported their scores on the math section of either the SAT or ACT test; 20 participants from the study sample chose not to report this information. We used this information to calculate a percentile score for each participant, as a measure of mathematics ability. The set of items about interest in and attitudes about math were drawn from several different sources; these items were included for exploratory purposes, as we did not have specific a priori hypotheses about interest, attitudes, and strategy change. Therefore, we do not consider these data in this report.

##### Debriefing

After completing the packet, participants were thanked and thoroughly debriefed. Recall that some participants in the feedback condition had solved the second pretest problem correctly, but were told their answer was incorrect; these participants received a more extensive debriefing, so as to make sure they understood the deception and understood that their pretest response was, in fact, correct.

#### Coding and exclusion criteria

The need for cognition scale was scored using the procedure described in Cacioppo et al. (1984). To assess participants’ confidence in the pretest strategy (i.e., the summation strategy for all participants in the study sample), we averaged their confidence ratings for the two pretest items.

Two research assistants coded participants’ written work for the strategy or strategies used on each problem. These codes indicated whether participants successfully used one of the common strategies (summation, Gauss, or area) and whether the participants attempted to use one of these strategies. Participants were considered to have attempted a strategy if their written work showed evidence of using the strategy, but they made an error in implementing the strategy. For instance, if a participant drew a trapezoid diagram and labeled it correctly, but made an error in calculating the area, this was considered an attempt to use the area strategy.

To assess reliability of strategy coding, data from 74 participants (23% of the full sample) were double coded; agreement on strategy codes was 85% (*N* = 370 items). These strategy codes were used to determine whether participants met the criterion for inclusion in the study sample (i.e., using or attempting only the summation strategy at pretest), whether participants adopted or attempted the area strategy at posttest, and whether participants adopted or attempted the Gauss strategy at posttest. Note that, because there were multiple posttest problems, and because participants could use multiple strategies on individual problems, these posttest categories were not mutually exclusive.

### Results and discussion

#### Preliminaries and overview of analysis

Our primary dependent variable was whether participants used or attempted to use the target strategy—in this study, the area strategy. Throughout the results, we use the term *strategy adoption* to refer to all attempts to use the target strategy, including both incomplete or incorrect attempts to use the strategy and successful use of the strategy.

In the control condition, in which participants were not exposed to any strategies, no participants generated the target (area) strategy or the alternative (Gauss) strategy. This was true even for the participants who received negative feedback about their answers to the second pretest problem. In other words, learning that their pretest strategy yielded an incorrect answer did not lead participants to spontaneously generate the target strategy when they were not exposed to it. Because there was no adoption or generation of this strategy in the control condition, we do not consider the control condition further. Instead we focus on the differing rates of strategy adoption in the three conditions in which participants were exposed to alternative strategies.

We used logistic regression to predict whether participants adopted the area strategy on *at least one* of the posttest problems. Recall that our hypothesis concerned the likelihood of adopting a new strategy when also exposed to one’s own strategy (in this case, the summation strategy). Thus, we used nonorthogonal contrasts (with the target-with-own-strategy condition as the reference group) to compare the likelihood of adopting the area strategy in the target-with-own-strategy condition to the likelihood of adopting the target area strategy in the target-in-isolation and target-with-other-novel-strategy conditions. In addition to exposure condition, we also tested whether receiving negative feedback about the final pretest item increased participants’ likelihood of adopting the target strategy. We also examined whether learners’ need for cognition and confidence in their pretest strategies were associated with adopting the target strategy.

To identify the best-fitting statistical model, we started with a model that included feedback condition, exposure condition, need for cognition, and confidence ratings for the pretest (summation) strategy, as well as all two- and three-way interactions involving these factors; we did not include the four-way interaction because we believed it would not be interpretable. We used a model comparison approach to identify the model of best fit, by comparing nested models that did versus did not include each of the terms, starting with higher-order effects and then moving to lower-order effects. We then winnowed the model down by deleting non-significant higher order effects, one at a time. This approach yielded a final model in which all of the predictors were significant or were part of a significant interaction. In light of past work showing associations between mathematics ability and strategy adoption for constant change problems (e.g., Brown, 2018), we made the a priori decision to control for mathematics ability (as measured by self-reported ACT or SAT math scores) in all models. Participants in the exposure conditions who did not report ACT or SAT scores (*N* = 14) were dropped from these analyses.

#### Adopting the target strategy

**Fig. 4:**
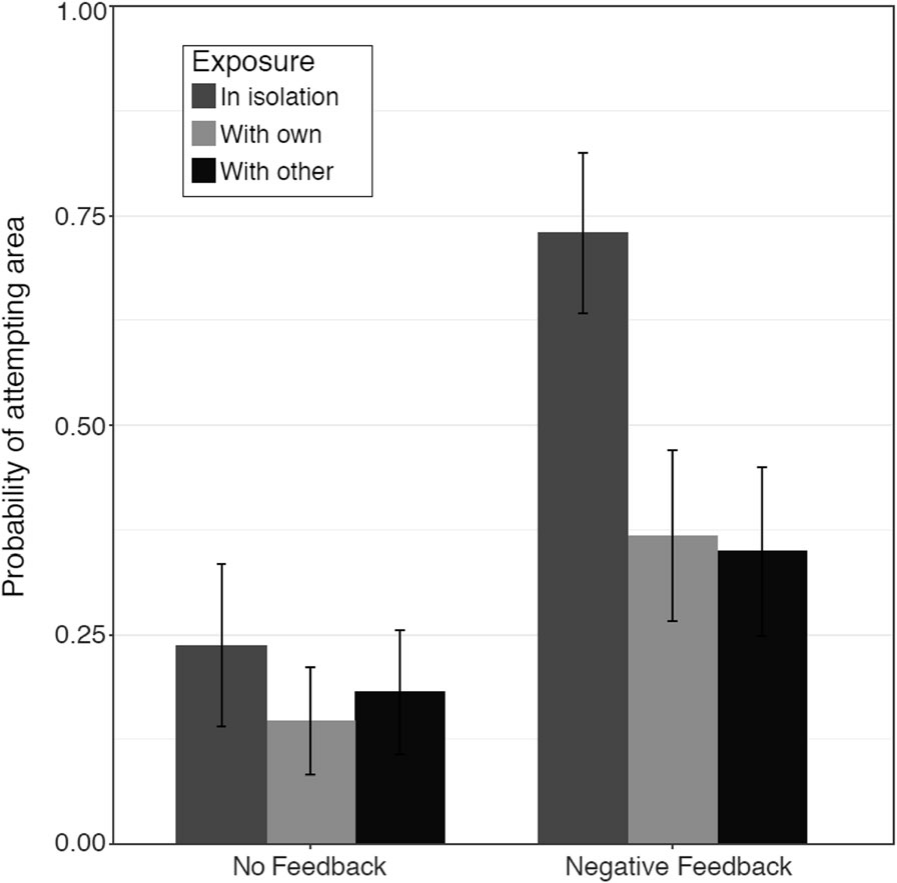
The probability of adopting the target (area) strategy in each condition in Study 1. The control condition is not depicted because no participants in the control condition adopted the area strategy. Error bars display standard errors

The proportion of participants in each condition who adopted the target strategy is presented in Fig. 4. The best-fitting model for this outcome measure included main effects of feedback condition, exposure condition, need for cognition, and confidence in the pretest (summation) strategy, and no interactions (Table 1). In line with our hypothesis about the role of negative feedback, particularly when it is given privately, participants were more likely to adopt the target area strategy when they were given negative feedback on the second pretest problem than when they received no feedback (*b* = 1.41, *F*(1, 143) = 11.82, *p* < 0.001). Thus, explicit negative feedback fostered adoption of the target strategy.

**Table 1:** Best-fitting model for predicting adoption of the area strategy in Study 1

Predictors	*b*	*SE*	*F*	*p*	*OR*
Exposure (with own vs. two novel)	0.07	0.47	0.02	.888	1.07
Exposure (with own vs. in isolation)	1.12	0.48	5.35	.021	3.06
Feedback	1.41	0.41	11.82	< .001	4.09
Need for cognition	1.41	0.59	5.73	.017	4.08
Confidence	−0.57	0.28	4.20	.040	0.57
SAT/ACT percentile	0.05	0.03	3.22	.073	1.05

As hypothesized, participants were more likely to adopt the target strategy when they encountered it in isolation than when they encountered it with their pretest (summation) strategy (*b* = 1.12, *F*(1, 143) = 5.35, *p* = 0.02; Fig. 4). This difference was greater among participants who received negative feedback, but the interaction of feedback and the in-isolation vs with-own contrast was not significant (*F*(1, 141) = 0.96, *p* = 0.33), so it was not retained in the final model. Participants were equally likely to adopt the target strategy when they encountered it with their own strategy and with another novel strategy (Gauss; *b* = 0.07, *F*(1, 143) = 0.02, *p* = 0.89).

There were also significant effects of learner characteristics. As predicted, participants with higher need for cognition scores were more likely to adopt the target strategy (*b* = 1.41, *F*(1, 143) = 5.73, *p* = 0.02). Also as predicted, participants who were highly confident in their pretest (summation) strategy were less likely to adopt the target (area) strategy than were participants who were less confident (*b* = − 0.57, *F*(1, 143) = 4.20, *p* = 0.04). Finally, there was a non-significant trend that participants with higher ACT or SAT scores were more likely to adopt the target strategy (*b* = 0.05, *F*(1, 143) = 3.22, *p* = 0.07).

These results suggest that many factors affect the likelihood of adopting the target strategy. In line with our predictions, telling participants that their answer to the final pretest problem was incorrect increased the likelihood of their adopting the target strategy at posttest. The context in which the target strategy was presented also influenced participants’ likelihood of adopting it. Participants were more likely to adopt the target strategy when they encountered it in isolation than when they were exposed to it along with their own strategy. This finding offers some support for our hypothesis that exposure to a target strategy in the context of one’s own strategy may decrease the likelihood of strategy change. In addition, learners with lower confidence in their existing strategies and higher need for cognition were more likely to adopt the target strategy. Thus, both contextual factors and learner characteristics were associated with patterns of strategy change.

Participants were equally likely to adopt the target strategy when exposed to the target and another novel strategy than when they were exposed to the target strategy and their own strategy. Thus, it may be that exposure to the target strategy in the context of *any* other strategy decreases participants’ likelihood of adopting it. On the other hand, it is possible that more participants who were exposed to two novel strategies chose to adopt a novel strategy, but many of them chose to adopt the Gauss strategy, rather than the area strategy.

#### Adopting either of two novel strategies

To explore this possibility, we further analyzed the data for the *target-with-other-novel-strategy* condition. For this analysis, participants were given two scores, one indicating whether they adopted the area strategy and one indicating whether they adopted the Gauss strategy at posttest (note that it was possible for participants to use both). We used a mixed-effects logistic regression to predict adopting a new strategy. This model included feedback condition, confidence, need for cognition, and mathematics ability as between-subjects predictors and strategy type (area or Gauss) as a within-subjects predictor. All two- and three-way interactions as well as by-subject random intercepts and by-subject random slopes for strategy were also included in the model. We followed the procedure described above to identify the best-fitting model.

**Fig. 5:**
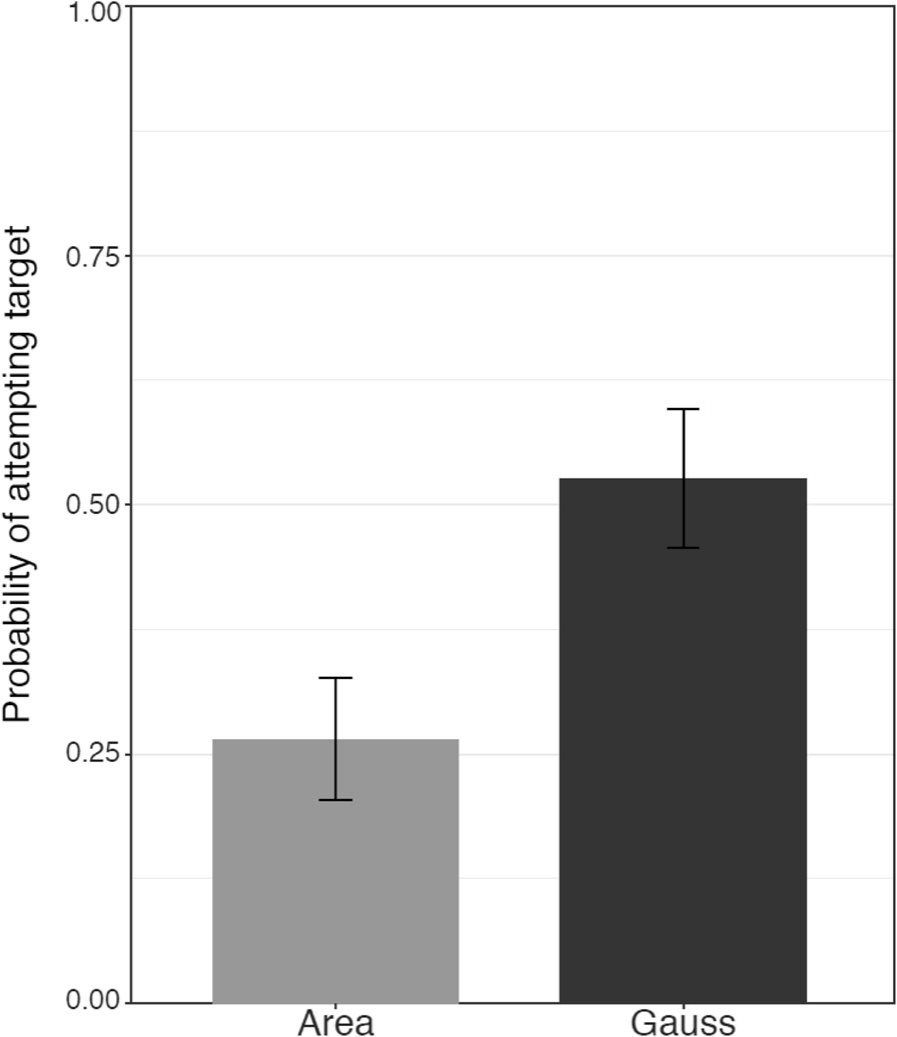
The probability of adopting the area strategy or the Gauss strategy when exposed to both. *Error bars* display standard errors

When exposed to both the area and the Gauss strategies, participants were more likely to adopt the Gauss strategy than the area strategy (*b* = − 11.22, *χ*^*2*^(1, *N* = 59) = 21.03, *p* < 0.001), as seen in Fig. 5. No other effects were significant, which is unsurprising given the small number of participants in the *target-with-other-novel-strategy* condition.

The difference in adoption of the area and Gauss strategies could be due to differences in how participants view the strategies. In a previous study, Brown et al. (2018) asked participants to rate the summation, area, and Gauss strategies on six different dimensions. They found that participants’ ratings cohered into two correlated factors, which they interpreted as the *intuitiveness* of the strategy and the *efficiency* of the strategy. Participants in that study rated the Gauss strategy and the area strategy as similar in efficiency, but they rated the Gauss strategy as more intuitive than the area strategy. It is possible that this difference in how learners view the two strategies influenced their likelihood of adopting the two strategies. Thus, it is possible that patterns of strategy adoption in Study 1 are due to our using the less intuitive area strategy as the target. To test this possibility, in Study 2, we examined patterns of adoption for the more intuitive Gauss strategy.

## Study 2

The results of Study 1 were in line with our hypothesis that the context in which a target strategy is presented to learners influences the likelihood that the strategy will be adopted. However, we wanted to make sure these results were not specific to the particular target strategy we used, the area strategy. To address this possibility, in Study 2, we tested whether the same factors affect adoption of the Gauss strategy.

In particular, we focus on the target-with-own-strategy and the target-in-isolation conditions. In brief, the goal of Study 2 was to investigate whether the results of Study 1 generalize to a different target strategy (Gauss) or whether the observed effects depend on the specific strategy. Using data from both studies, we examine whether the effects of exposure to a novel target strategy, presented either in isolation or accompanied by one’s own strategy, vary depending on the specific strategy (area or Gauss).

### Method

#### Participants

One hundred and sixty undergraduate students from a large Midwestern university participated for extra credit in their introductory psychology course. Of these 160 participants, 103 identified as female, 55 identified as male, and 2 chose not to report their gender. Additionally, 117 described themselves as white, 20 as Asian or Asian-American, 2 as black or African-American, 9 as Hispanic or Latinx, 5 as bi- or multi-racial, and 2 as some other race or ethnicity; 5 chose not to report race or ethnicity. The average age of the participants was 18.83 years (*SD* = 1.02 years).

Three participants (2%) were excluded because they did not follow directions. As in Study 1, our analyses focused on the participants who used only the summation strategy at pretest. Forty-five participants (28%) were excluded from analyses because they used a strategy other than the summation strategy on the pretest. Thus, the study sample consisted of 112 participants.

#### Design

The study used a 2 (feedback: none or negative) × 2 (strategy exposure context: target-in-isolation or target-with-own-strategy) between-subjects design. Participants were randomly assigned to one of the four conditions.

#### Coding

Strategies were coded in the same way and by the same coders as in Study 1. Data from 39 participants (24% of the full sample) were coded by two independent coders; agreement on strategy codes was 87% (*N* = 195). These codes were used by the same two researchers as in Study 1 to determine which participants fit the inclusion criterion of using or attempting only the summation strategy at pretest, whether participants adopted or attempted the area strategy at posttest, and whether participants adopted or attempted the Gauss strategy at posttest.

### Results and discussion

#### Preliminaries and overview of data analysis

To address the research questions, we combined data from Studies 1 and 2. We used data from both studies from the conditions in which people were exposed to a target strategy in isolation and to a target strategy with their own strategy. We used logistic regression to predict the probability that participants adopted the target strategy (i.e., attempted it on at least one problem), and we conducted the data analysis using the same process as in Study 1. Participants who did not report ACT or SAT scores (a total of 15 participants, including 6 from the Study 2 sample and 9 from the Study 1 sample) were dropped from the analyses.

Given that the data were drawn from two different studies (Study 1 with area as the target strategy and Study 2 with Gauss as the target strategy), participants were not randomly assigned to target strategy condition (although they were randomly assigned to condition within each study). However, all participants were drawn from the same population (i.e., students in an introductory psychology course at the same university). To ensure that the samples were comparable in other ways, we tested for differences in participants’ SAT/ACT percentile scores (*t*(305) = 0.42, not significant), need for cognition scores (*t*(328) = 0.50, not significant), and confidence in their pretest strategies (*t*(331) = 1.54, not significant) and found no differences.

To address whether the effects of exposure to a novel target strategy vary depending on the specific strategy, we started with an initial model that included target strategy (area or Gauss), exposure (in isolation or with own), feedback (negative feedback or no feedback), need for cognition scores, and pretest confidence in the summation strategy as predictors, controlling for mathematics ability. In our initial model, we included the four-way interactions that included target strategy, all three-way interactions, and all lower-level effects. We used the same process as in Study 1 to identify the best-fitting model.

#### Adopting the target strategy

The model of best fit included main effects of strategy, feedback, exposure, confidence, and need for cognition, as well as two-way interactions of strategy by feedback and exposure by feedback, and the three-way interaction of strategy, need for cognition, and confidence. The main effects of feedback and need for cognition were as in Study 1, so we do not discuss them further here. The effect of pretest confidence in the summation strategy, observed in Study 1, was not significant as a main effect in the best-fitting model (*p* = 0.06; Table 2), though confidence was involved in a higher-order interaction, as discussed below.

**Table 2:** Predictors of adoption of target strategy for Study 2

Predictors	*b*	*SE*	*F*	*p*	*OR*
Strategy (area vs Gauss)	−0.68	0.34	3.95	.047	0.51
Exposure (with own vs in isolation)	−0.48	0.33	2.13	.145	0.62
Feedback	0.93	0.34	7.48	.006	2.52
Need for cognition	1.12	0.52	4.56	.033	3.06
Confidence	−0.45	0.24	3.57	.059	0.64
Strategy × Feedback	1.81	0.68	7.16	.007	6.12
Exposure × Feedback	−1.69	0.66	6.57	.010	0.18
Strategy × Need for cognition	2.02	1.04	3.76	.052	7.57
Strategy × Confidence	0.78	0.45	3.00	.084	2.18
Need for cognition × Confidence	−0.79	0.64	1.49	.222	0.46
Strategy × Need for cognition × Confidence	−2.86	1.29	4.89	.027	0.06
SAT/ACT percentile	0.01	0.02	0.22	.636	1.01

**Fig. 6:**
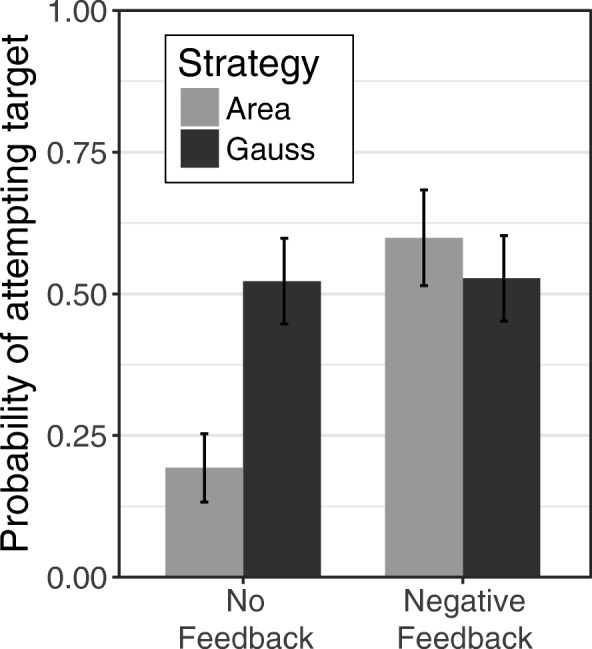
The probability of adopting the area and Gauss strategies with no feedback and with negative feedback about the pretest (summation) strategy. Error bars display standard errors

Overall, and as in the analysis of the two-novel-strategies condition of Study 1, participants were less likely to adopt the area strategy than the Gauss strategy (*b* = − 0.68, *F*(1, 189) = 3.95, *p* = 0.047). This main effect was qualified by a significant interaction between strategy and feedback (*b* = 1.81, *F*(1, 189) = 7.16, *p* = 0.007). As seen in Fig. 6, participants tended to adopt the Gauss strategy regardless of whether or not they received negative feedback, whereas participants were much more likely to adopt the area strategy if they received negative feedback than if they did not. Thus, the effectiveness of negative feedback depended on the specific strategy in question.

**Fig. 7:**
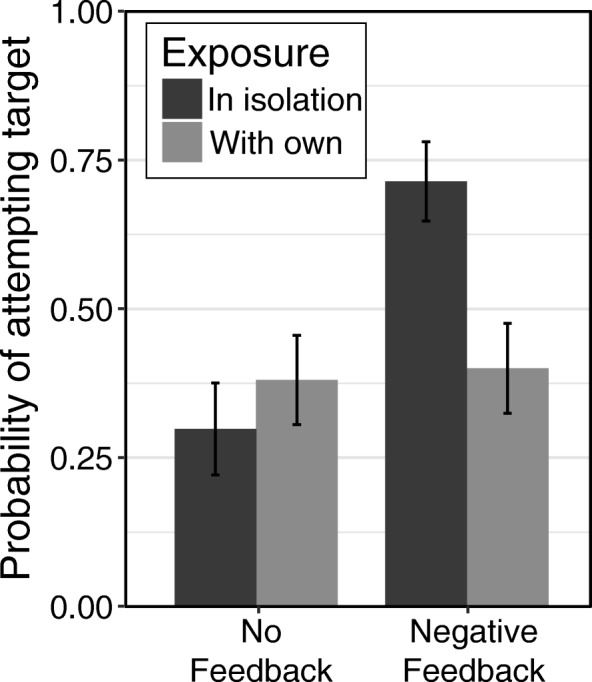
The probability of adopting the target strategy in the no feedback and negative feedback conditions, when it was presented in isolation vs with participants' own current strategy. *Error bars* display standard errors

The interaction of exposure context and feedback was also significant (*b* = − 1.69, *F*(1, 189) = 6.57, *p* = 0.01; Fig. 7). Among participants who did not receive feedback, there was no difference in the likelihood of adopting the target strategy between those who encountered the strategy in isolation and those who encountered it with their own strategy (*p* = 0.44). However, participants who received negative feedback were more likely to adopt the target strategy when they encountered it in isolation than when they encountered it with their own strategy (*p* = 0.003). This latter finding supports our hypothesis that knowing that someone else used the same strategy might be perceived as implicit feedback that a strategy is correct, which could undermine the effects of negative feedback. In addition, this finding offers support for the hypothesis that such implicit, positive feedback about one’s current strategy might decrease the likelihood of strategy change. It is worth noting that this effect was *not* moderated by the specific strategy, suggesting that the effects of encountering a novel strategy in isolation versus with one’s own strategy were similar for both the area and the Gauss strategies. However, it is also worth noting that this exposure context–feedback interaction did not emerge in Study 1.

**Fig. 8:**
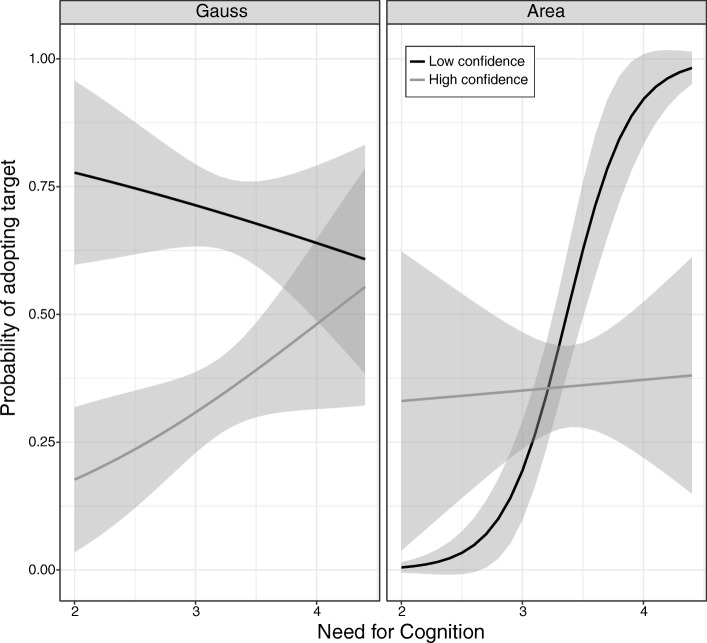
Three-way interaction between strategy, confidence, and need for cognition

There was also a significant three-way interaction of strategy, confidence, and need for cognition (*b* = − 2.86, *F*(1, 189) = 4.89, *p* = 0.03; Fig. 8). Among participants who encountered the Gauss strategy (left panel), participants with higher levels of confidence in their pretest (summation) strategy were less likely to adopt the Gauss strategy (*F*(1, 189) = 8.54, *p* = 0.003). This simple effect did not depend on need for cognition (*F*(1, 189) = 1.00, *p* = 0.32), and the simple effect of need for cognition also was not significant (*F*(1, 189) = 0.04, *p* = 0.85). In contrast, for participants who encountered the area strategy (right panel), the association between confidence in their pretest strategy and adopting the area strategy depended on participants’ level of need for cognition (*F*(1, 189) = 3.92, *p* = 0.048). Among those with lower confidence in their pretest (summation) strategy (Fig. 8, right panel, black line), there was a striking effect of need for cognition (*p* = 0.017), but among those with high confidence in their pretest strategy (Fig. 8, right panel, grey line), there was no effect of need for cognition (*p* = 0.28).

These results suggest that adoption of a target strategy depends on the strategy itself. In general, people may be more inclined to adopt strategies that seem highly intuitive (Brown et al., 2018) than strategies that seem relatively unintuitive. However, people who might not otherwise adopt the less intuitive strategy can be motivated to do so by telling them that their current answers are wrong. In addition, the likelihood of adopting particular strategies also depends on individual differences, such as need for cognition and confidence in one’s current strategy.

## General discussion

### Empirical summary

These studies support a complex, dynamic view of the process of strategy change. Participants’ likelihood of adopting a new strategy depended not only on whether they were exposed to that strategy, but also on the context in which that strategy was presented (in isolation, with their current strategy, or with another novel strategy). Additionally, receiving negative feedback increased participants’ likelihood of adopting a new strategy, but only if participants were exposed to at least one new strategy and were not also exposed to their current strategy. Furthermore, the specific strategy also mattered. Participants were more likely to adopt the more intuitive Gauss strategy than the less intuitive area strategy. When participants received explicit, negative feedback, however, these strategy differences were not observed.

We also found that learner characteristics, both stable and transitory, were associated with patterns of strategy change. In line with the dynamic view, their role depended on the particular strategy to which participants were exposed. When participants encountered the area strategy—which is complicated and was rated low in intuitiveness in prior work (Brown et al., 2018)—both participants’ need for cognition and their confidence in their prior strategy mattered. When they encountered the more intuitive Gauss strategy, need for cognition was not associated with patterns of strategy change.

The effects of the exposure context show that strategy change is most likely when learners encounter a target strategy in isolation; this effect appears to be independent of the specific strategy, at least for the strategies we tested. When participants were exposed to only one strategy, they were relatively likely to adopt it. When they were exposed to two strategies, they were likely to adopt one of them. However, when participants were exposed to their own strategy along with a new strategy, they were less likely to adopt the new strategy. This supports the hypothesis that telling learners that someone else used their existing strategy serves as implicit positive feedback.

It appears that implicit positive feedback of this sort can counteract the effects of explicit negative feedback. Overall, receiving negative feedback increased the likelihood that learners adopted a new strategy—however, the simple effect of feedback was significant only for participants who encountered the novel strategy in isolation, and not for participants who learned that someone else had also used their current strategy (Fig. 7).

### Contributions and implications for theoretical accounts of strategy change

One important contribution of the current studies is to demonstrate the importance of strategy availability in processes of strategy change. It is noteworthy that no participants in the control condition, in which participants were not exposed to any novel strategies, started using the Gauss or area strategy at posttest—even though half of them received negative feedback about their current strategy. This finding suggests that the strategy change observed in the other conditions, in which participants were exposed to one or more novel strategies, was not due simply to within-item strategy variability. If it were, we would expect some strategy change in the control condition as well. Instead, the lack of change in the control condition relative to the other conditions suggests that having a viable alternative strategy at one’s disposal greatly increases the likelihood of strategy change.

Relatedly, our findings have implications for understanding the role of feedback in strategy change. In this research, feedback was important for strategy change, as indicated by the large main effect of feedback for learners who received exposure to new strategies. However, the effects of feedback varied. As discussed above, negative feedback alone was not enough to cause participants in the control condition to generate either the Gauss strategy or the area strategy; learners need an available alternative. Thus, in considering whether feedback will be effective in a particular case, it is important to consider whether the learners have alternative strategies upon which to draw. It is also important to consider whether aspects of the context may implicitly support the “status quo”. For participants who received exposure to a novel strategy, the effects of direct, negative feedback on strategy adoption depended on whether the strategy was presented in isolation or along with participants’ existing strategy. Solvers who learned that another student had also used their current strategy tended to maintain that strategy, even in the face of negative feedback. Other kinds of experiences may similarly function as implicit positive feedback.

Many theoretical and computational models of strategy change incorporate feedback as a key driver of strategy change (e.g., the Strategy Selection Learning Theory, Rieskamp & Otto, 2006; the Represent-Construct-Choose-Learn model, Lovett & Schunn, 1999; the Adaptive Strategy Choice Model, Siegler & Shipley, 1995). In these models, learners obtain information about whether strategies are successful by implementing them and receiving feedback about their accuracy. On each problem, learners select strategies on the basis of their knowledge about the strategies’ success rates (in some cases, along with some random error). Positive feedback about a strategy increases the likelihood that that strategy will be selected on a subsequent trial, and negative feedback decreases that likelihood. These models assume that the effects of feedback are fairly straightforward; however, our findings suggest that the effects of feedback are more varied and dependent on other factors. Future models of strategy change may need to incorporate mechanisms that allow for varying effects of feedback, including variations that depend on features of the context.

The present work also demonstrates that individual differences in traits, such as need for cognition, can influence learners’ strategy choices, and that the effects of such individual differences may depend on the particular strategy in question. In this research, adoption of the area strategy depended on participants’ level of need for cognition, but adoption of the Gauss strategy did not. There are presumably other individual differences that we did not measure that could also be relevant to strategy adoption; for example, visuo-spatial ability might influence participants’ willingness to attempt the diagram-based area strategy.

Existing models of strategy choice are fairly limited in their treatment of individual differences. Some work has involved varying model parameters to simulate variations in the efficiency of learning and the speed with which attention is shifted (e.g., in the Strategy Choice and Discovery Simulation model for mathematical inversion problems, SCADS*, Siegler & Araya, 2005). Some models incorporate “confidence criteria”—a threshold that an association between a problem and an answer must surpass in order for the system to report the answer rather than apply a back-up strategy—and allow for variation in the stringency of these criteria across learners (e.g., the Distribution of Associations model (Siegler & Shrager, 1984), and its successor, the Adaptive Strategy Choice Model (Siegler & Shipley, 1995), for arithmetic). However, no models of strategy choice have considered traits such as need for cognition. Future research in this area will need to more deeply consider the role of individual differences in strategy choices.

The present findings converge with other work to highlight the importance of metacognitive factors in understanding patterns of strategy change (e.g., Crowley, Shrager, & Siegler, 1997; Geurten & Lemaire, 2017; Geurten, Lemaire, & Meulemans, 2018; Waters & Kunnmann, 2010). Past research has suggested that people rely on metacognitive judgments to guide their strategy selection; for example, in research using an arithmetic estimation task, Geurten and Lemaire (2018) found that when adults had low confidence that they had selected the optimal strategy on a given problem, they were more likely to select the optimal strategy on the next problem. Geurten and Lemaire (2018) suggested that participants’ lack of confidence served as a “warning signal” that they should pay more attention to information that could be useful for strategy selection. Along similar lines, we found in Study 1 that participants who were less confident in the summation strategy at pretest were more likely to try the area strategy, if exposed to it, and we found in Study 2 that such participants were more likely to try the Gauss strategy, if exposed to it. Further, participants in Study 2 who were less confident in the summation strategy at pretest and who were also high in need for cognition were also more likely to try the novel area strategy, if exposed to it. Taken together, these findings suggest that participants with low confidence in their pretest strategies attended carefully to information that was potentially relevant to strategy selection—in line with the findings of Geurten and Lemaire (2018).

Existing models of strategy change do not fully specify the role of metacognitive factors. Although many models propose that learners track information about strategies’ success rates, to our knowledge, no existing models incorporate information about learners’ confidence in particular *strategies* (as opposed to, e.g., strengths of associations between problems and solutions). Likewise, no existing models incorporate information about the types of strategy characteristics that individual learners may value. Although some models—the Represent-Construct-Choose-Learn model in particular (Lovett & Schunn, 1999)—highlight the importance of people’s representations of the problems, most existing models do not consider how people view or represent *strategies*.

Our current and past work (Brown et al., 2018) suggests that learners may have strong intuitions about strategies when they first encounter them, even without information about their success rates. In some cases, learners may dislike or prefer a particular strategy without ever having attempted the strategy and without having received feedback on its effectiveness. In this regard, it is worth noting that two computational models of strategy choice, SCADS (for addition problems, Shrager & Siegler, 1998) and SCADS* (for inversion problems, Siegler & Araya, 2005), do explicitly incorporate metacognitive mechanisms for evaluating novel strategies. These models evaluate potential new strategies using “goal sketch filters”, which embody learners’ knowledge about the goals a particular strategy must meet, and thereby enable learners to avoid illegitimate strategies. In the current studies, we did not offer participants illegitimate strategies, so this type of metacognitive mechanism was not applicable. However, our findings suggest that models of strategy change may need to include a mechanism that selects among novel legitimate approaches, in addition to a mechanism that filters out illegitimate strategies. One important goal for future work is to understand what features of strategies learners attend to when deciding whether or not to adopt a possible strategy, and how those features may vary with other individual differences, such as prior knowledge and attitudes toward the domain.

It seems likely that individual differences in learners’ views of the intuitiveness and efficiency of alternative strategies, though not directly measured in this study, are at least partially responsible for the differing rates of adoption of the two target strategies. As one example, learners who are high in need for cognition may place a high value on the intuitiveness of a potential strategy, whereas learners who are low in need for cognition may place a greater value on the efficiency of an alternative. Thus, strategy change depends not only on characteristics of strategies and characteristics of learners, but also on learners’ evaluations of the characteristics of strategies, which themselves may vary depending on characteristics of the learners! Further research is needed to elucidate these complex interactions. In addition, the present findings suggest that the context in which learners encounter new strategies might influence their representations of those strategies. Both learners’ initial impressions of novel strategies and the importance of the context in which novel strategies are encountered are details that are yet to be accounted for in models of strategy change.

The current work highlights the complexity of the mechanisms that underlie strategy adoption. Even factors that seem simple and straightforward—such as offering negative feedback about an existing strategy or demonstrating an alternative strategy—do not operate in the same way in all contexts, for all learners, or for all strategies. The same information is interpreted differently depending on whether it is provided in isolation or in the context of the learner’s current strategy, and depending on both stable and transitory individual characteristics. The specifics of the proffered alternative strategy matter as well—though perhaps not in the same way for all learners.

Indeed, our findings highlight the need for a comprehensive account of strategy change that can integrate multiple factors at different levels of analysis (see Alibali et al., 2019, for discussion of this point). One approach would be to conceptualize strategy change as due to an accumulation of factors that either push people toward change (*risk* factors) or protect them against change (*protective* factors). Models of this sort—termed *cumulative risk models*—have been used by scholars and policy makers to predict a range of outcomes in other domains, including health and risk behavior (see, e.g., Price & Hyde, 2009; Raviv, Taussig, Culhane, & Garrido, 2010). Another approach would be to conceptualize strategy change as a dynamic system (see, e.g., Spencer, Austin, & Schutte, 2012; Thelen & Smith, 1998) with a diverse set of potential “control parameters”, including contextual and individual factors, which may operate over different timescales. Within a dynamic system, these factors may come together in non-additive or nonlinear ways to influence whether the system remains in a particular state (i.e., maintains its current strategy) or shifts to a new state (i.e., adopts a new approach). Future work is needed to specify and test such comprehensive models, and fine-grained empirical data—like the data presented herein—could be used to validate potential models or to adjudicate among potential models.

### Implications for education

In educational settings, teachers sometimes encourage students to change their strategies for solving problems. For example, teachers may wish for students to use formal algebraic strategies when solving linear equations, rather than informal, arithmetic strategies (see Koedinger, Alibali, & Nathan, 2008). Teachers may present new strategies in direct instruction, or they may encourage students to work together to generate new strategies.

The current findings suggest that the context in which learners encounter new strategies is likely to matter. Novel strategies presented in isolation may be more readily adopted than novel strategies presented in the context of common but non-optimal strategies. These findings may have implications for how strategies should be presented and discussed in classroom settings. If a teacher’s goal is to encourage students to shift to a particular novel approach, it may not be helpful to describe or consider existing approaches—at least without additional guidance.

Research on comparison and conceptual change suggests that comparing and contrasting multiple strategies can improve performance and understanding (e.g., Rittle-Johnson & Star, 2011; Star, Rittle-Johnson, & Durkin, 2016). In the current studies, participants did not receive any instruction to compare the strategies, and the strategies were presented one at a time (rather than simultaneously), which may have discouraged comparison. Thus, although exposure to multiple strategies may be beneficial when students are given instructions about how to engage with the strategies, when given neither information about the quality of the strategies nor instructions to compare, exposure to additional strategies may hinder the adoption of a target strategy.

The present findings further suggest that learners may not be equally likely to adopt new strategies presented in instruction. Learners who are more confident in their prior strategies may be less likely to shift their approaches, and learners who are low in need for cognition may also be resistant to change. However, these effects may also depend on the specific strategy under consideration, and whether it is highly intuitive or less so. Teachers should be mindful of these individual differences and how they may influence students’ receptivity to instruction about new strategies.

### Limitations and future directions

This research considered several different factors that influence patterns of strategy adoption. However, there are many influential factors beyond those considered here. Some studies have documented sequential effects in people’s strategy choices; the strategy selected on a particular problem may vary depending on the strategy used on the immediately preceding problems (e.g., Lemaire, 2016). Other studies have demonstrated effects of problem features, such as the format in which a problem is presented (e.g., as an equation or a word problem; Koedinger et al., 2008) or the presence of a diagram or illustration (Cooper, Sidney, & Alibali, 2018). Other individual difference factors besides those we considered here are also likely to be relevant, either on their own or in interaction with contextual or problem-specific factors. Some potentially relevant individual differences are prior knowledge, visuospatial abilities, and working memory capacity.

In this research, we used a relatively coarse measure of strategy change: whether or not participants ever attempted the target strategy. It could also be informative to investigate other, more fine-grained measures of strategy change. For instance, some researchers have investigated adults’ and children’s abilities to switch between strategies on a single item (e.g., Ardiale & Lemaire, 2012, 2013a, 2013b). In the present studies, participants did at times use more than one strategy on a single item; however, because participants wrote their answers on paper, we were unable to determine the order in which participants attempted these strategies, and whether participants ever switched strategies mid-execution. Other methods, such as talk-aloud protocols, could shed more light on within-item strategy variability and change. Identifying factors that lead learners to abandon a strategy and attempt another on a single item would further our understanding of how people select strategies.

## Conclusions

Learning about a new strategy does not occur in a vacuum. Many factors matter, including aspects of the context in which the strategy is presented, characteristics of the learner, and features of the strategy in question. This work identified several factors that are influential in strategy adoption, both on their own and in combination. Overall, learners were more likely to adopt a novel strategy when it was presented on its own than when it was presented in the context of the learners' own strategy. However, this effect depended on whether learners also received explicit negative feedback. Learners were more likely to adopt one of the proffered strategies (Gauss) than the other (area), but this effect was moderated by learner characteristics, including need for cognition and confidence in their existing strategy. The findings suggest that strategy adoption depends on the confluence of many factors. To fully understand patterns of strategy change will require consideration of the context in which a target strategy is introduced, characteristics of the learner, and characteristics of the strategy itself.

## Footnotes

1 This strategy is named after the mathematician Carl Friedrich Gauss. According to legend, his elementary school teacher assigned Gauss to complete a daunting task: add all the numbers from 1 to 100. However, Gauss generated this strategy and was able to solve the problem quickly, much to the surprise of his teacher.

2 The correct answer to this problem is 175. However, when using the summation strategy, people sometimes settle on an incorrect constant and number of intervals, leading to an incorrect answer. In the problem for which participants received feedback (the second pretest problem), participants often used a constant of 6 for 7 intervals, yielding the incorrect solution of 200, rather than using a constant of 7 for 6 intervals, yielding the correct solution of 175.
